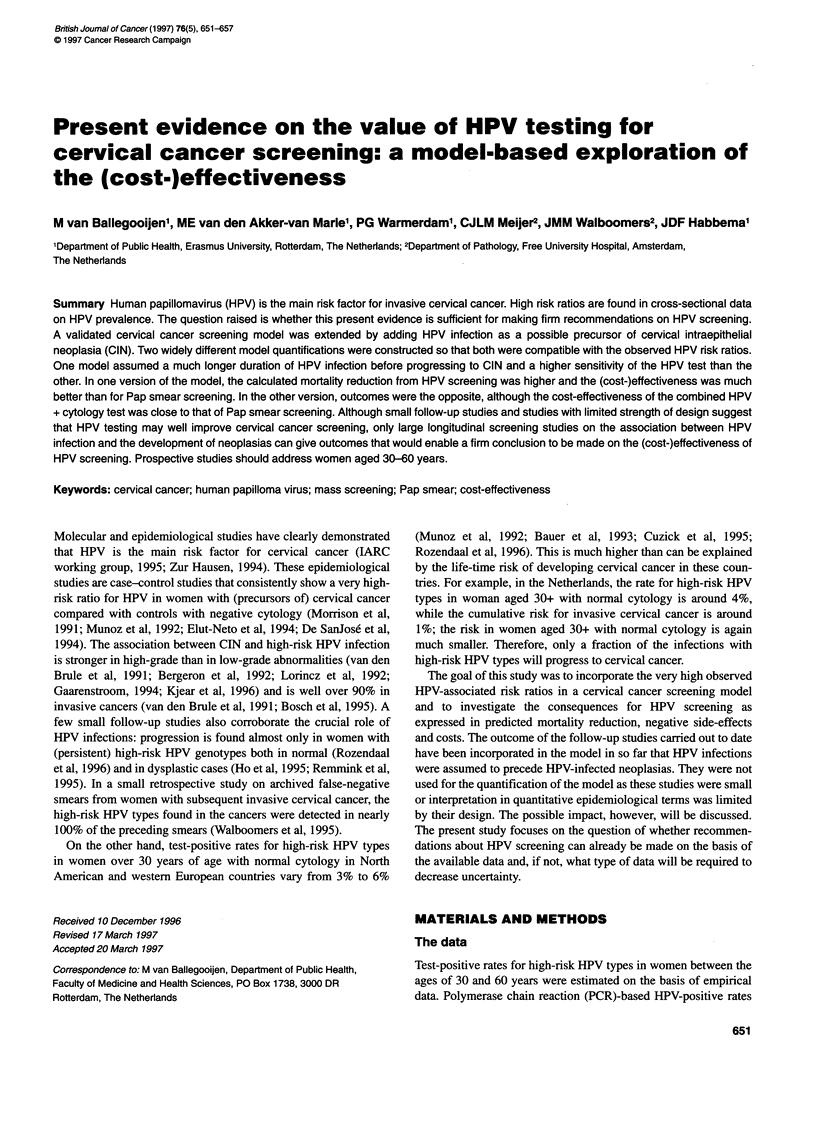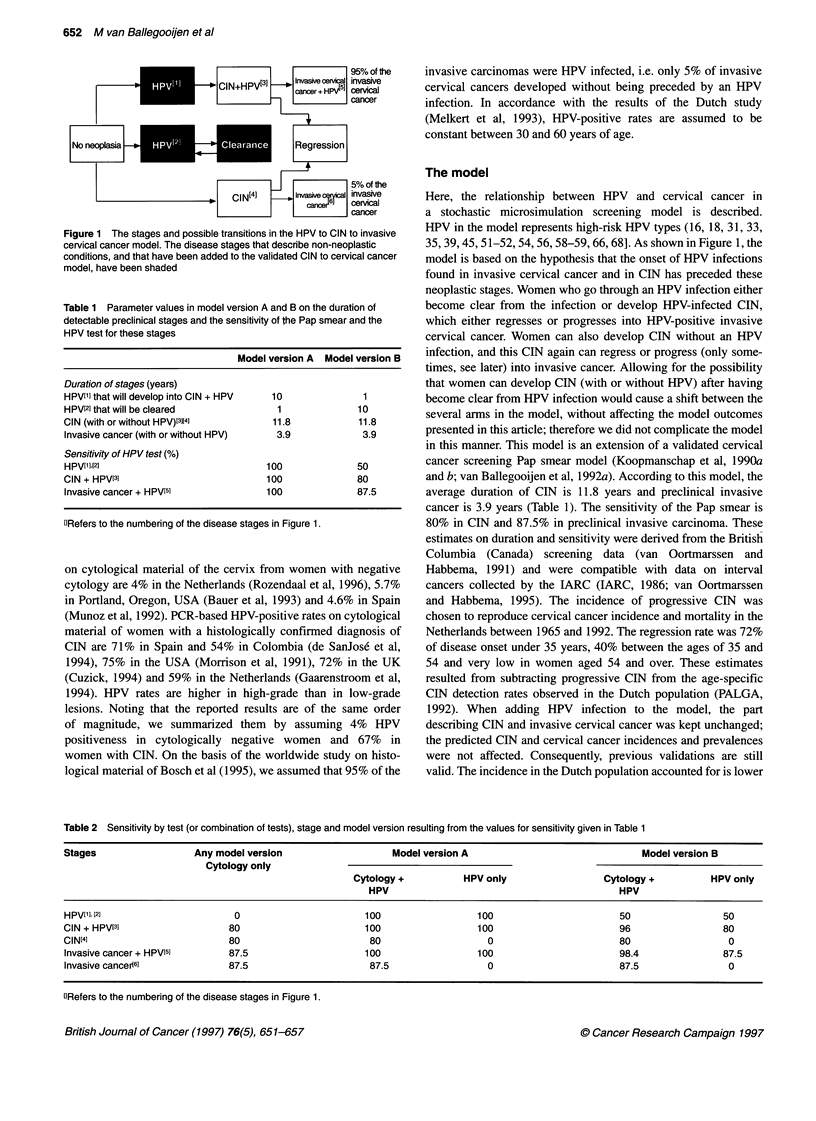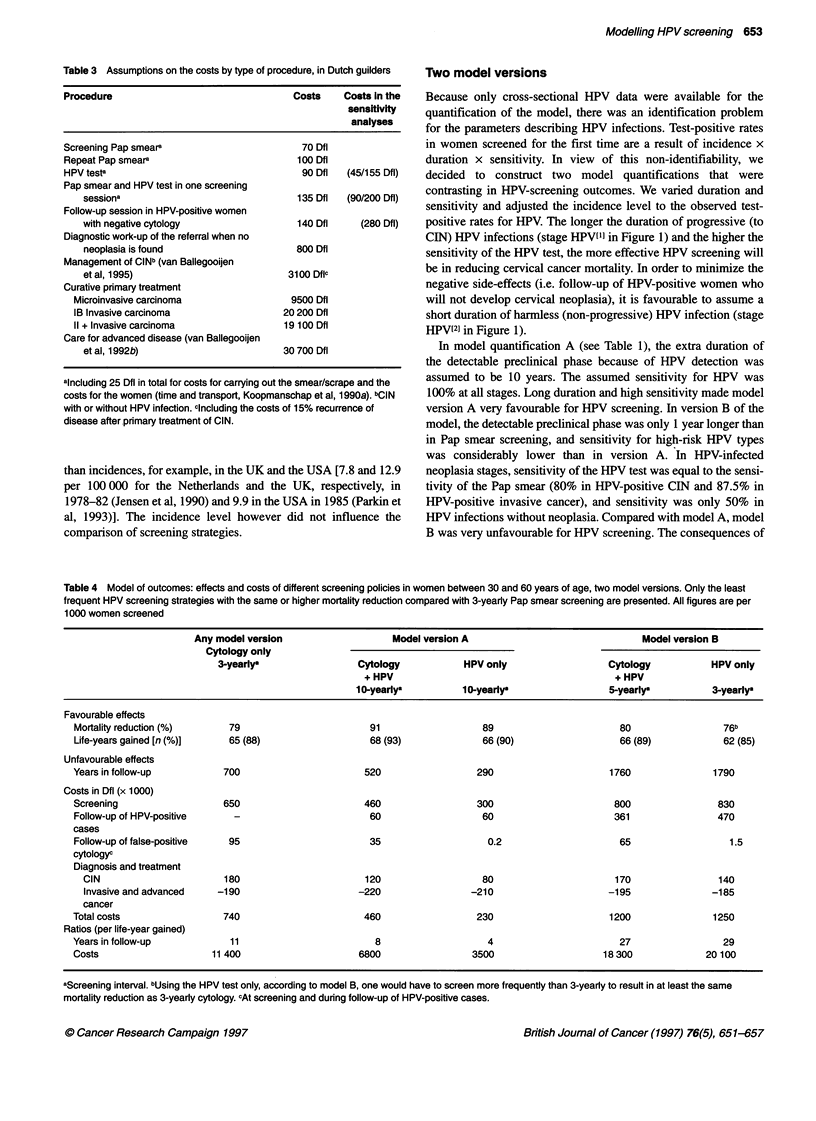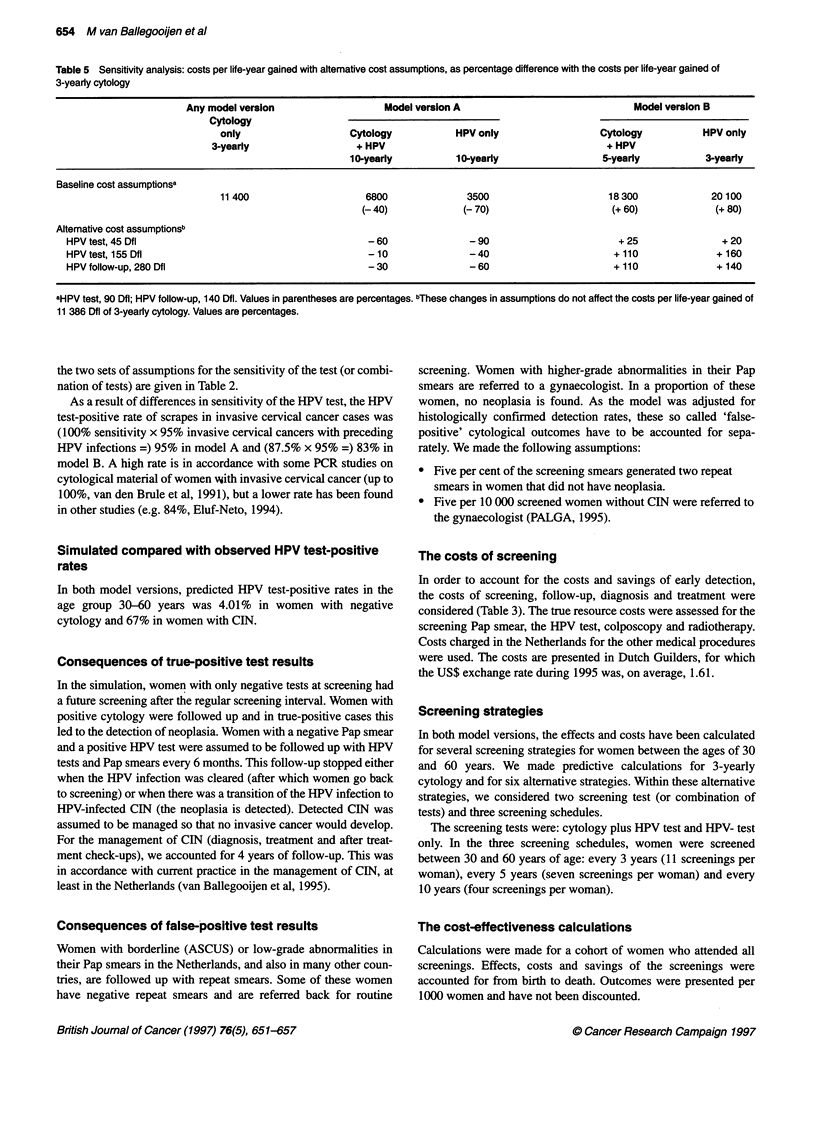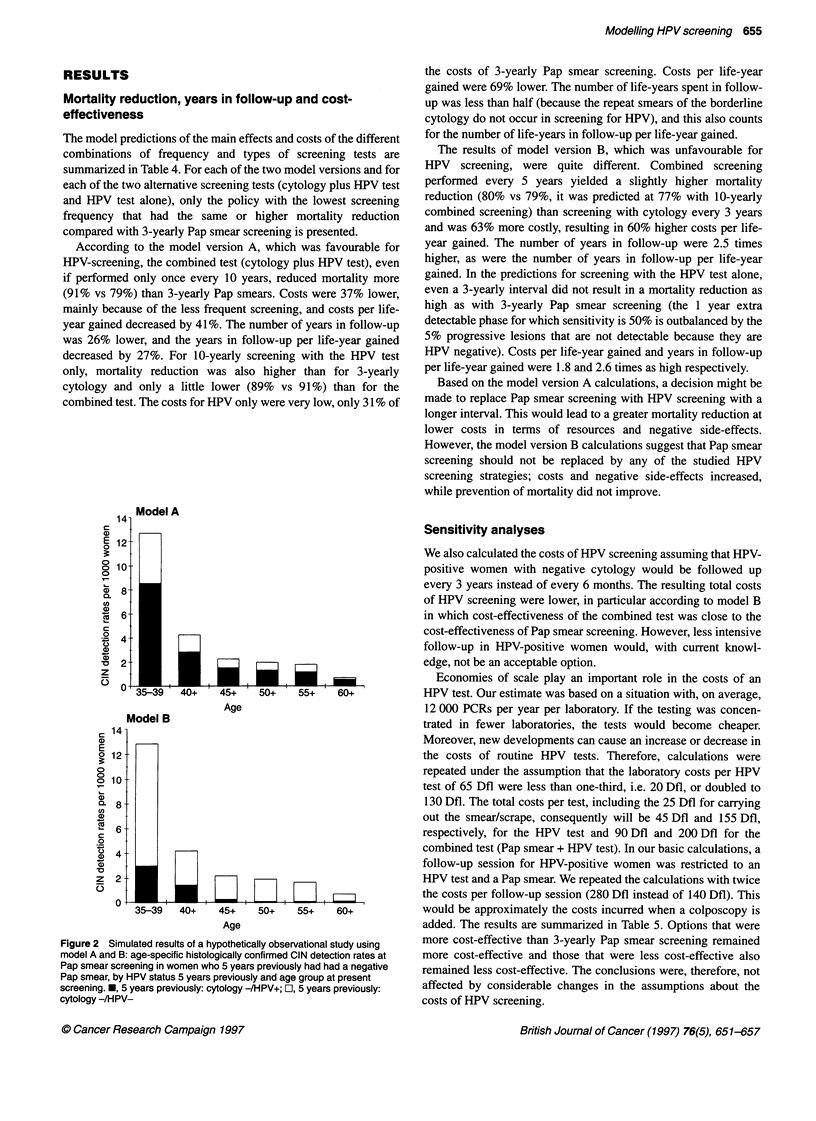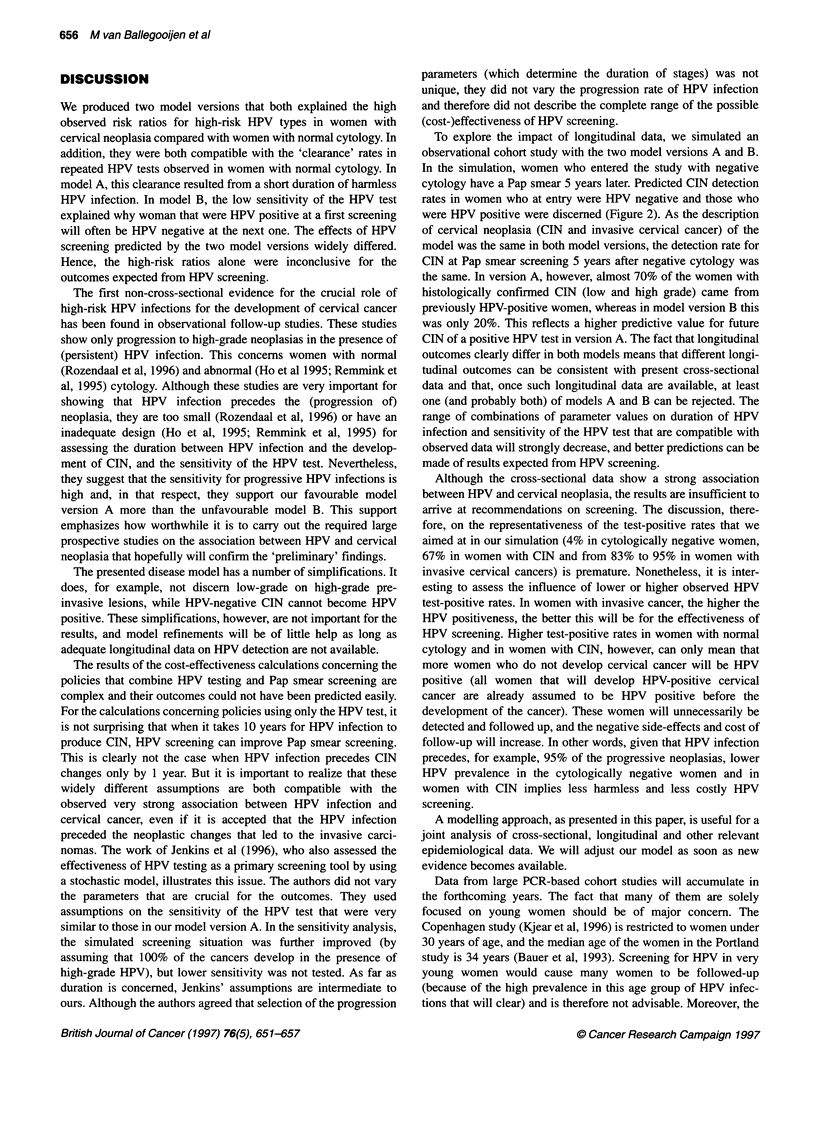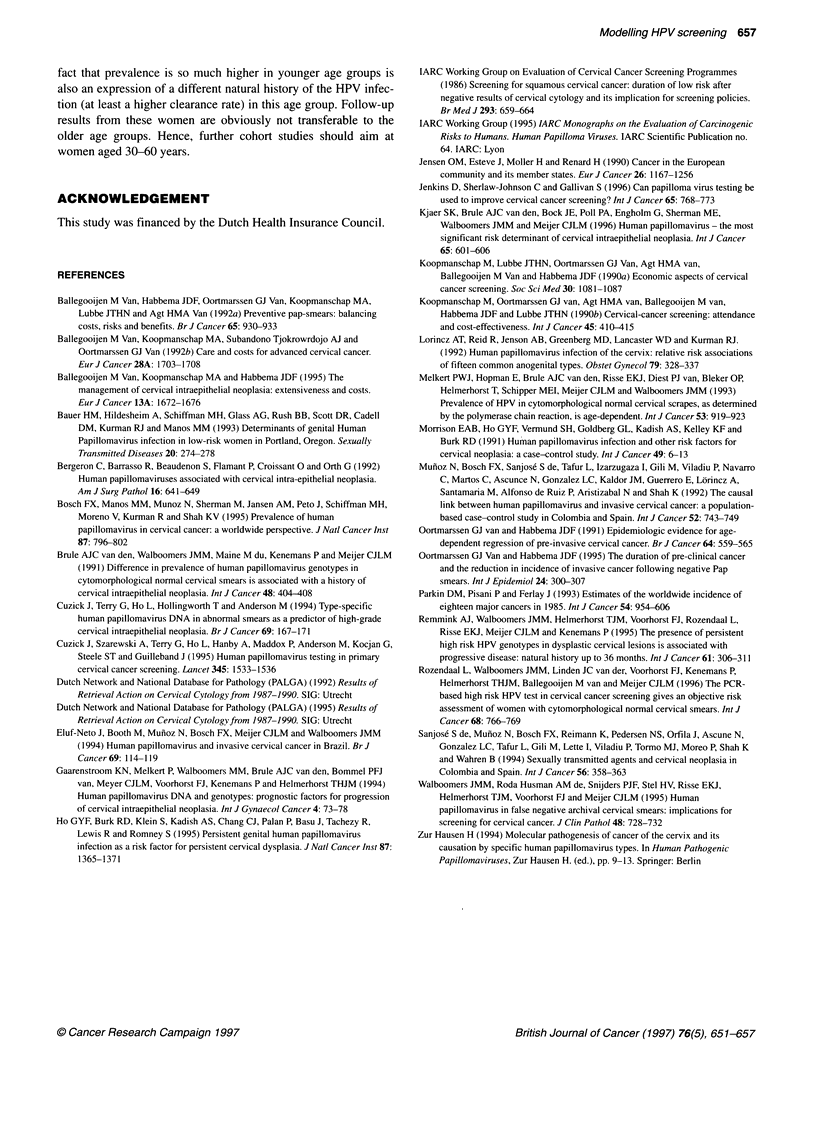# Present evidence on the value of HPV testing for cervical cancer screening: a model-based exploration of the (cost-)effectiveness.

**DOI:** 10.1038/bjc.1997.440

**Published:** 1997

**Authors:** M. van Ballegooijen, M. E. van den Akker-van Marle, P. G. Warmerdam, C. J. Meijer, J. M. Walboomers, J. D. Habbema

**Affiliations:** Department of Public Health, Erasmus University, Rotterdam, The Netherlands.

## Abstract

Human papillomavirus (HPV) is the main risk factor for invasive cervical cancer. High risk ratios are found in cross-sectional data on HPV prevalence. The question raised is whether this present evidence is sufficient for making firm recommendations on HPV screening. A validated cervical cancer screening model was extended by adding HPV infection as a possible precursor of cervical intraepithelial neoplasia (CIN). Two widely different model quantifications were constructed so that both were compatible with the observed HPV risk ratios. One model assumed a much longer duration of HPV infection before progressing to CIN and a higher sensitivity of the HPV test than the other. In one version of the model, the calculated mortality reduction from HPV screening was higher and the (cost-)effectiveness was much better than for Pap smear screening. In the other version, outcomes were the opposite, although the cost-effectiveness of the combined HPV + cytology test was close to that of Pap smear screening. Although small follow-up studies and studies with limited strength of design suggest that HPV testing may well improve cervical cancer screening, only large longitudinal screening studies on the association between HPV infection and the development of neoplasias can give outcomes that would enable a firm conclusion to be made on the (cost-)effectiveness of HPV screening. Prospective studies should address women aged 30-60 years.


British Journal of Cancer (1997) 76(5), 651–657
© 1997 Cancer Research Campaign

### Present evidence on the value of HPV testing for cervical cancer screening: a model-based exploration of the (cost-)effectiveness

**M van Ballegooijen[1], ME van den Akker-van Marle[1], PG Warmerdam[1], CJLM Meijer[2], JMM Walboomers[2], JDF Habbema[1]**

[1]Department of Public Health, Erasmus University, Rotterdam, The Netherlands; [2]Department of Pathology, Free University Hospital, Amsterdam, The Netherlands

**Summary** Human papillomavirus (HPV) is the main risk factor for invasive cervical cancer. High risk ratios are found in cross-sectional data on HPV prevalence. The question raised is whether this present evidence is sufficient for making firm recommendations on HPV screening. A validated cervical cancer screening model was extended by adding HPV infection as a possible precursor of cervical intraepithelial neoplasia (CIN). Two widely different model quantifications were constructed so that both were compatible with the observed HPV risk ratios. One model assumed a much longer duration of HPV infection before progressing to CIN and a higher sensitivity of the HPV test than the other. In one version of the model, the calculated mortality reduction from HPV screening was higher and the (cost-)effectiveness was much better than for Pap smear screening. In the other version, outcomes were the opposite, although the cost-effectiveness of the combined HPV + cytology test was close to that of Pap smear screening. Although small follow-up studies and studies with limited strength of design suggest that HPV testing may well improve cervical cancer screening, only large longitudinal screening studies on the association between HPV infection and the development of neoplasias can give outcomes that would enable a firm conclusion to be made on the (cost-)effectiveness of HPV screening. Prospective studies should address women aged 30–60 years.

**Keywords:** cervical cancer; human papilloma virus; mass screening; Pap smear; cost-effectiveness

Molecular and epidemiological studies have clearly demonstrated that HPV is the main risk factor for cervical cancer (IARC working group, 1995; Zur Hausen, 1994). These epidemiological studies are case–control studies that consistently show a very high-risk ratio for HPV in women with (precursors of) cervical cancer compared with controls with negative cytology (Morrison et al, 1991; Munoz et al, 1992; Elut-Neto et al, 1994; De SanJosé et al, 1994). The association between CIN and high-risk HPV infection is stronger in high-grade than in low-grade abnormalities (van den Brule et al, 1991; Bergeron et al, 1992; Lorincz et al, 1992; Gaarenstroom, 1994; Kjear et al, 1996) and is well over 90% in invasive cancers (van den Brule et al, 1991; Bosch et al, 1995). A few small follow-up studies also corroborate the crucial role of HPV infections: progression is found almost only in women with (persistent) high-risk HPV genotypes both in normal (Rozendaal et al, 1996) and in dysplastic cases (Ho et al, 1995; Remmink et al, 1995). In a small retrospective study on archived false-negative smears from women with subsequent invasive cervical cancer, the high-risk HPV types found in the cancers were detected in nearly 100% of the preceding smears (Walboomers et al, 1995).

On the other hand, test-positive rates for high-risk HPV types in women over 30 years of age with normal cytology in North American and western European countries vary from 3% to 6% (Munoz et al, 1992; Bauer et al, 1993; Cuzick et al, 1995; Rozendaal et al, 1996). This is much higher than can be explained by the life-time risk of developing cervical cancer in these countries. For example, in the Netherlands, the rate for high-risk HPV types in woman aged 30+ with normal cytology is around 4%, while the cumulative risk for invasive cervical cancer is around 1%; the risk in women aged 30+ with normal cytology is again much smaller. Therefore, only a fraction of the infections with high-risk HPV types will progress to cervical cancer.

The goal of this study was to incorporate the very high observed HPV-associated risk ratios in a cervical cancer screening model and to investigate the consequences for HPV screening as expressed in predicted mortality reduction, negative side-effects and costs. The outcome of the follow-up studies carried out to date have been incorporated in the model in so far that HPV infections were assumed to precede HPV-infected neoplasias. They were not used for the quantification of the model as these studies were small or interpretation in quantitative epidemiological terms was limited by their design. The possible impact, however, will be discussed. The present study focuses on the question of whether recommendations about HPV screening can already be made on the basis of the available data and, if not, what type of data will be required to decrease uncertainty.

*Received 10 December 1996*
*Revised 17 March 1997*
*Accepted 20 March 1997*

*Correspondence to:* M van Ballegooijen, Department of Public Health, Faculty of Medicine and Health Sciences, PO Box 1738, 3000 DR Rotterdam, The Netherlands

### MATERIALS AND METHODS

### The data

Test-positive rates for high-risk HPV types in women between the ages of 30 and 60 years were estimated on the basis of empirical data. Polymerase chain reaction (PCR)-based HPV-positive rates

**Figure 1** The stages and possible transitions in the HPV to CIN to invasive cervical cancer model. The disease stages that describe non-neoplastic conditions, and that have been added to the validated CIN to cervical cancer model, have been shaded

**Table 1** Parameter values in model version A and B on the duration of detectable preclinical stages and the sensitivity of the Pap smear and the HPV test for these stages

| | Model version A | Model version B |
|---|---|---|
| *Duration of stages* (years) | | |
| HPV[1] that will develop into CIN + HPV | 10 | 1 |
| HPV[2] that will be cleared | 1 | 10 |
| CIN (with or without HPV)[3][4] | 11.8 | 11.8 |
| Invasive cancer (with or without HPV) | 3.9 | 3.9 |
| *Sensitivity of HPV test* (%) | | |
| HPV[1],[2] | 100 | 50 |
| CIN + HPV[3] | 100 | 80 |
| Invasive cancer + HPV[5] | 100 | 87.5 |

Refers to the numbering of the disease stages in Figure 1.

on cytological material of the cervix from women with negative cytology are 4% in the Netherlands (Rozendaal et al, 1996), 5.7% in Portland, Oregon, USA (Bauer et al, 1993) and 4.6% in Spain (Munoz et al, 1992). PCR-based HPV-positive rates on cytological material of women with a histologically confirmed diagnosis of CIN are 71% in Spain and 54% in Colombia (de SanJosé et al, 1994), 75% in the USA (Morrison et al, 1991), 72% in the UK (Cuzick, 1994) and 59% in the Netherlands (Gaarenstroom et al, 1994). HPV rates are higher in high-grade than in low-grade lesions. Noting that the reported results are of the same order of magnitude, we summarized them by assuming 4% HPV positiveness in cytologically negative women and 67% in women with CIN. On the basis of the worldwide study on histological material of Bosch et al (1995), we assumed that 95% of the invasive carcinomas were HPV infected, i.e. only 5% of invasive cervical cancers developed without being preceded by an HPV infection. In accordance with the results of the Dutch study (Melkert et al, 1993), HPV-positive rates are assumed to be constant between 30 and 60 years of age.

### The model

Here, the relationship between HPV and cervical cancer in a stochastic microsimulation screening model is described. HPV in the model represents high-risk HPV types (16, 18, 31, 33, 35, 39, 45, 51–52, 54, 56, 58–59, 66, 68]. As shown in Figure 1, the model is based on the hypothesis that the onset of HPV infections found in invasive cervical cancer and in CIN has preceded these neoplastic stages. Women who go through an HPV infection either become clear from the infection or develop HPV-infected CIN, which either regresses or progresses into HPV-positive invasive cervical cancer. Women can also develop CIN without an HPV infection, and this CIN again can regress or progress (only sometimes, see later) into invasive cancer. Allowing for the possibility that women can develop CIN (with or without HPV) after having become clear from HPV infection would cause a shift between the several arms in the model, without affecting the model outcomes presented in this article; therefore we did not complicate the model in this manner. This model is an extension of a validated cervical cancer screening Pap smear model (Koopmanschap et al, 1990*a* and *b*; van Ballegooijen et al, 1992*a*). According to this model, the average duration of CIN is 11.8 years and preclinical invasive cancer is 3.9 years (Table 1). The sensitivity of the Pap smear is 80% in CIN and 87.5% in preclinical invasive carcinoma. These estimates on duration and sensitivity were derived from the British Columbia (Canada) screening data (van Oortmarssen and Habbema, 1991) and were compatible with data on interval cancers collected by the IARC (IARC, 1986; van Oortmarssen and Habbema, 1995). The incidence of progressive CIN was chosen to reproduce cervical cancer incidence and mortality in the Netherlands between 1965 and 1992. The regression rate was 72% of disease onset under 35 years, 40% between the ages of 35 and 54 and very low in women aged 54 and over. These estimates resulted from subtracting progressive CIN from the age-specific CIN detection rates observed in the Dutch population (PALGA, 1992). When adding HPV infection to the model, the part describing CIN and invasive cervical cancer was kept unchanged; the predicted CIN and cervical cancer incidences and prevalences were not affected. Consequently, previous validations are still valid. The incidence in the Dutch population accounted for is lower

**Table 2** Sensitivity by test (or combination of tests), stage and model version resulting from the values for sensitivity given in Table 1

| Stages | Any model version Cytology only | Model version A | | Model version B | |
|---|---|---|---|---|---|
| | | Cytology + HPV | HPV only | Cytology + HPV | HPV only |
| HPV[1], [2] | 0 | 100 | 100 | 50 | 50 |
| CIN + HPV[3] | 80 | 100 | 100 | 96 | 80 |
| CIN[4] | 80 | 80 | 0 | 80 | 0 |
| Invasive cancer + HPV[5] | 87.5 | 100 | 100 | 98.4 | 87.5 |
| Invasive cancer[6] | 87.5 | 87.5 | 0 | 87.5 | 0 |

Refers to the numbering of the disease stages in Figure 1.

© Cancer Research Campaign 1997

Table 3 Assumptions on the costs by type of procedure, in Dutch guilders

| Procedure | Costs | Costs in the sensitivity analyses |
|---|---|---|
| Screening Pap smear[a] | 70 Dfl | |
| Repeat Pap smear[a] | 100 Dfl | |
| HPV test[a] | 90 Dfl | (45/155 Dfl) |
| Pap smear and HPV test in one screening session[a] | 135 Dfl | (90/200 Dfl) |
| Follow-up session in HPV-positive women with negative cytology | 140 Dfl | (280 Dfl) |
| Diagnostic work-up of the referral when no neoplasia is found | 800 Dfl | |
| Management of CIN[b] (van Ballegooijen et al, 1995) | 3100 Dfl[c] | |
| Curative primary treatment | | |
| Microinvasive carcinoma | 9500 Dfl | |
| IB Invasive carcinoma | 20 200 Dfl | |
| II + Invasive carcinoma | 19 100 Dfl | |
| Care for advanced disease (van Ballegooijen et al, 1992*b*) | 30 700 Dfl | |

[a]Including 25 Dfl in total for costs for carrying out the smear/scrape and the costs for the women (time and transport, Koopmanschap et al, 1990*a*). [b]CIN with or without HPV infection. [c]Including the costs of 15% recurrence of disease after primary treatment of CIN.

than incidences, for example, in the UK and the USA [7.8 and 12.9 per 100 000 for the Netherlands and the UK, respectively, in 1978–82 (Jensen et al, 1990) and 9.9 in the USA in 1985 (Parkin et al, 1993)]. The incidence level however did not influence the comparison of screening strategies.

### Two model versions

Because only cross-sectional HPV data were available for the quantification of the model, there was an identification problem for the parameters describing HPV infections. Test-positive rates in women screened for the first time are a result of incidence × duration × sensitivity. In view of this non-identifiability, we decided to construct two model quantifications that were contrasting in HPV-screening outcomes. We varied duration and sensitivity and adjusted the incidence level to the observed test-positive rates for HPV. The longer the duration of progressive (to CIN) HPV infections (stage HPV[1] in Figure 1) and the higher the sensitivity of the HPV test, the more effective HPV screening will be in reducing cervical cancer mortality. In order to minimize the negative side-effects (i.e. follow-up of HPV-positive women who will not develop cervical neoplasia), it is favourable to assume a short duration of harmless (non-progressive) HPV infection (stage HPV[2] in Figure 1).

In model quantification A (see Table 1), the extra duration of the detectable preclinical phase because of HPV detection was assumed to be 10 years. The assumed sensitivity for HPV was 100% at all stages. Long duration and high sensitivity made model version A very favourable for HPV screening. In version B of the model, the detectable preclinical phase was only 1 year longer than in Pap smear screening, and sensitivity for high-risk HPV types was considerably lower than in version A. In HPV-infected neoplasia stages, sensitivity of the HPV test was equal to the sensitivity of the Pap smear (80% in HPV-positive CIN and 87.5% in HPV-positive invasive cancer), and sensitivity was only 50% in HPV infections without neoplasia. Compared with model A, model B was very unfavourable for HPV screening. The consequences of

Table 4 Model of outcomes: effects and costs of different screening policies in women between 30 and 60 years of age, two model versions. Only the least frequent HPV screening strategies with the same or higher mortality reduction compared with 3-yearly Pap smear screening are presented. All figures are per 1000 women screened

| | Any model version Cytology only 3-yearly[a] | Model version A | | Model version B | |
|---|---|---|---|---|---|
| | | Cytology + HPV 10-yearly[a] | HPV only 10-yearly[a] | Cytology + HPV 5-yearly[a] | HPV only 3-yearly[a] |
| Favourable effects | | | | | |
| Mortality reduction (%) | 79 | 91 | 89 | 80 | 76[b] |
| Life-years gained [*n* (%)] | 65 (88) | 68 (93) | 66 (90) | 66 (89) | 62 (85) |
| Unfavourable effects | | | | | |
| Years in follow-up | 700 | 520 | 290 | 1760 | 1790 |
| Costs in Dfl (× 1000) | | | | | |
| Screening | 650 | 460 | 300 | 800 | 830 |
| Follow-up of HPV-positive cases | – | 60 | 60 | 361 | 470 |
| Follow-up of false-positive cytology[c] | 95 | 35 | 0.2 | 65 | 1.5 |
| Diagnosis and treatment | | | | | |
| CIN | 180 | 120 | 80 | 170 | 140 |
| Invasive and advanced cancer | –190 | –220 | –210 | –195 | –185 |
| Total costs | 740 | 460 | 230 | 1200 | 1250 |
| Ratios (per life-year gained) | | | | | |
| Years in follow-up | 11 | 8 | 4 | 27 | 29 |
| Costs | 11 400 | 6800 | 3500 | 18 300 | 20 100 |

[a]Screening interval. [b]Using the HPV test only, according to model B, one would have to screen more frequently than 3-yearly to result in at least the same mortality reduction as 3-yearly cytology. [c]At screening and during follow-up of HPV-positive cases.

© Cancer Research Campaign 1997

Table 5 Sensitivity analysis: costs per life-year gained with alternative cost assumptions, as percentage difference with the costs per life-year gained of 3-yearly cytology

| | Any model version | Model version A | | Model version B | |
|---|---|---|---|---|---|
| | Cytology only 3-yearly | Cytology + HPV 10-yearly | HPV only 10-yearly | Cytology + HPV 5-yearly | HPV only 3-yearly |
| Baseline cost assumptions[a] | 11 400 | 6800 (– 40) | 3500 (– 70) | 18 300 (+ 60) | 20 100 (+ 80) |
| Alternative cost assumptions[b] | | | | | |
| HPV test, 45 Dfl | | – 60 | – 90 | + 25 | + 20 |
| HPV test, 155 Dfl | | – 10 | – 40 | + 110 | + 160 |
| HPV follow-up, 280 Dfl | | – 30 | – 60 | + 110 | + 140 |

[a]HPV test, 90 Dfl; HPV follow-up, 140 Dfl. Values in parentheses are percentages. [b]These changes in assumptions do not affect the costs per life-year gained of 11 386 Dfl of 3-yearly cytology. Values are percentages.

the two sets of assumptions for the sensitivity of the test (or combination of tests) are given in Table 2.

As a result of differences in sensitivity of the HPV test, the HPV test-positive rate of scrapes in invasive cervical cancer cases was (100% sensitivity × 95% invasive cervical cancers with preceding HPV infections =) 95% in model A and (87.5% × 95% =) 83% in model B. A high rate is in accordance with some PCR studies on cytological material of women with invasive cervical cancer (up to 100%, van den Brule et al, 1991), but a lower rate has been found in other studies (e.g. 84%, Eluf-Neto, 1994).

### Simulated compared with observed HPV test-positive rates

In both model versions, predicted HPV test-positive rates in the age group 30–60 years was 4.01% in women with negative cytology and 67% in women with CIN.

### Consequences of true-positive test results

In the simulation, women with only negative tests at screening had a future screening after the regular screening interval. Women with positive cytology were followed up and in true-positive cases this led to the detection of neoplasia. Women with a negative Pap smear and a positive HPV test were assumed to be followed up with HPV tests and Pap smears every 6 months. This follow-up stopped either when the HPV infection was cleared (after which women go back to screening) or when there was a transition of the HPV infection to HPV-infected CIN (the neoplasia is detected). Detected CIN was assumed to be managed so that no invasive cancer would develop. For the management of CIN (diagnosis, treatment and after treatment check-ups), we accounted for 4 years of follow-up. This was in accordance with current practice in the management of CIN, at least in the Netherlands (van Ballegooijen et al, 1995).

### Consequences of false-positive test results

Women with borderline (ASCUS) or low-grade abnormalities in their Pap smears in the Netherlands, and also in many other countries, are followed up with repeat smears. Some of these women have negative repeat smears and are referred back for routine screening. Women with higher-grade abnormalities in their Pap smears are referred to a gynaecologist. In a proportion of these women, no neoplasia is found. As the model was adjusted for histologically confirmed detection rates, these so called 'false-positive' cytological outcomes have to be accounted for separately. We made the following assumptions:

- Five per cent of the screening smears generated two repeat smears in women that did not have neoplasia.
- Five per 10 000 screened women without CIN were referred to the gynaecologist (PALGA, 1995).

### The costs of screening

In order to account for the costs and savings of early detection, the costs of screening, follow-up, diagnosis and treatment were considered (Table 3). The true resource costs were assessed for the screening Pap smear, the HPV test, colposcopy and radiotherapy. Costs charged in the Netherlands for the other medical procedures were used. The costs are presented in Dutch Guilders, for which the US$ exchange rate during 1995 was, on average, 1.61.

### Screening strategies

In both model versions, the effects and costs have been calculated for several screening strategies for women between the ages of 30 and 60 years. We made predictive calculations for 3-yearly cytology and for six alternative strategies. Within these alternative strategies, we considered two screening test (or combination of tests) and three screening schedules.

The screening tests were: cytology plus HPV test and HPV- test only. In the three screening schedules, women were screened between 30 and 60 years of age: every 3 years (11 screenings per woman), every 5 years (seven screenings per woman) and every 10 years (four screenings per woman).

### The cost-effectiveness calculations

Calculations were made for a cohort of women who attended all screenings. Effects, costs and savings of the screenings were accounted for from birth to death. Outcomes were presented per 1000 women and have not been discounted.

 © Cancer Research Campaign 1997

### RESULTS

### Mortality reduction, years in follow-up and cost-effectiveness

The model predictions of the main effects and costs of the different combinations of frequency and types of screening tests are summarized in Table 4. For each of the two model versions and for each of the two alternative screening tests (cytology plus HPV test and HPV test alone), only the policy with the lowest screening frequency that had the same or higher mortality reduction compared with 3-yearly Pap smear screening is presented.

According to the model version A, which was favourable for HPV-screening, the combined test (cytology plus HPV test), even if performed only once every 10 years, reduced mortality more (91% vs 79%) than 3-yearly Pap smears. Costs were 37% lower, mainly because of the less frequent screening, and costs per life-year gained decreased by 41%. The number of years in follow-up was 26% lower, and the years in follow-up per life-year gained decreased by 27%. For 10-yearly screening with the HPV test only, mortality reduction was also higher than for 3-yearly cytology and only a little lower (89% vs 91%) than for the combined test. The costs for HPV only were very low, only 31% of the costs of 3-yearly Pap smear screening. Costs per life-year gained were 69% lower. The number of life-years spent in follow-up was less than half (because the repeat smears of the borderline cytology do not occur in screening for HPV), and this also counts for the number of life-years in follow-up per life-year gained.

The results of model version B, which was unfavourable for HPV screening, were quite different. Combined screening performed every 5 years yielded a slightly higher mortality reduction (80% vs 79%, it was predicted at 77% with 10-yearly combined screening) than screening with cytology every 3 years and was 63% more costly, resulting in 60% higher costs per life-year gained. The number of years in follow-up were 2.5 times higher, as were the number of years in follow-up per life-year gained. In the predictions for screening with the HPV test alone, even a 3-yearly interval did not result in a mortality reduction as high as with 3-yearly Pap smear screening (the 1 year extra detectable phase for which sensitivity is 50% is outbalanced by the 5% progressive lesions that are not detectable because they are HPV negative). Costs per life-year gained and years in follow-up per life-year gained were 1.8 and 2.6 times as high respectively.

Based on the model version A calculations, a decision might be made to replace Pap smear screening with HPV screening with a longer interval. This would lead to a greater mortality reduction at lower costs in terms of resources and negative side-effects. However, the model version B calculations suggest that Pap smear screening should not be replaced by any of the studied HPV screening strategies; costs and negative side-effects increased, while prevention of mortality did not improve.

**Figure 2** Simulated results of a hypothetically observational study using model A and B: age-specific histologically confirmed CIN detection rates at Pap smear screening in women who 5 years previously had had a negative Pap smear, by HPV status 5 years previously and age group at present screening. ■, 5 years previously: cytology –/HPV+; □, 5 years previously: cytology –/HPV–

### Sensitivity analyses

We also calculated the costs of HPV screening assuming that HPV-positive women with negative cytology would be followed up every 3 years instead of every 6 months. The resulting total costs of HPV screening were lower, in particular according to model B in which cost-effectiveness of the combined test was close to the cost-effectiveness of Pap smear screening. However, less intensive follow-up in HPV-positive women would, with current knowledge, not be an acceptable option.

Economies of scale play an important role in the costs of an HPV test. Our estimate was based on a situation with, on average, 12 000 PCRs per year per laboratory. If the testing was concentrated in fewer laboratories, the tests would become cheaper. Moreover, new developments can cause an increase or decrease in the costs of routine HPV tests. Therefore, calculations were repeated under the assumption that the laboratory costs per HPV test of 65 Dfl were less than one-third, i.e. 20 Dfl, or doubled to 130 Dfl. The total costs per test, including the 25 Dfl for carrying out the smear/scrape, consequently will be 45 Dfl and 155 Dfl, respectively, for the HPV test and 90 Dfl and 200 Dfl for the combined test (Pap smear + HPV test). In our basic calculations, a follow-up session for HPV-positive women was restricted to an HPV test and a Pap smear. We repeated the calculations with twice the costs per follow-up session (280 Dfl instead of 140 Dfl). This would be approximately the costs incurred when a colposcopy is added. The results are summarized in Table 5. Options that were more cost-effective than 3-yearly Pap smear screening remained more cost-effective and those that were less cost-effective also remained less cost-effective. The conclusions were, therefore, not affected by considerable changes in the assumptions about the costs of HPV screening.

© Cancer Research Campaign 1997

### DISCUSSION

We produced two model versions that both explained the high observed risk ratios for high-risk HPV types in women with cervical neoplasia compared with women with normal cytology. In addition, they were both compatible with the 'clearance' rates in repeated HPV tests observed in women with normal cytology. In model A, this clearance resulted from a short duration of harmless HPV infection. In model B, the low sensitivity of the HPV test explained why woman that were HPV positive at a first screening will often be HPV negative at the next one. The effects of HPV screening predicted by the two model versions widely differed. Hence, the high-risk ratios alone were inconclusive for the outcomes expected from HPV screening.

The first non-cross-sectional evidence for the crucial role of high-risk HPV infections for the development of cervical cancer has been found in observational follow-up studies. These studies show only progression to high-grade neoplasias in the presence of (persistent) HPV infection. This concerns women with normal (Rozendaal et al, 1996) and abnormal (Ho et al 1995; Remmink et al, 1995) cytology. Although these studies are very important for showing that HPV infection precedes the (progression of) neoplasia, they are too small (Rozendaal et al, 1996) or have an inadequate design (Ho et al, 1995; Remmink et al, 1995) for assessing the duration between HPV infection and the development of CIN, and the sensitivity of the HPV test. Nevertheless, they suggest that the sensitivity for progressive HPV infections is high and, in that respect, they support our favourable model version A more than the unfavourable model B. This support emphasizes how worthwhile it is to carry out the required large prospective studies on the association between HPV and cervical neoplasia that hopefully will confirm the 'preliminary' findings.

The presented disease model has a number of simplifications. It does, for example, not discern low-grade on high-grade pre-invasive lesions, while HPV-negative CIN cannot become HPV positive. These simplifications, however, are not important for the results, and model refinements will be of little help as long as adequate longitudinal data on HPV detection are not available.

The results of the cost-effectiveness calculations concerning the policies that combine HPV testing and Pap smear screening are complex and their outcomes could not have been predicted easily. For the calculations concerning policies using only the HPV test, it is not surprising that when it takes 10 years for HPV infection to produce CIN, HPV screening can improve Pap smear screening. This is clearly not the case when HPV infection precedes CIN changes only by 1 year. But it is important to realize that these widely different assumptions are both compatible with the observed very strong association between HPV infection and cervical cancer, even if it is accepted that the HPV infection preceded the neoplastic changes that led to the invasive carcinomas. The work of Jenkins et al (1996), who also assessed the effectiveness of HPV testing as a primary screening tool by using a stochastic model, illustrates this issue. The authors did not vary the parameters that are crucial for the outcomes. They used assumptions on the sensitivity of the HPV test that were very similar to those in our model version A. In the sensitivity analysis, the simulated screening situation was further improved (by assuming that 100% of the cancers develop in the presence of high-grade HPV), but lower sensitivity was not tested. As far as duration is concerned, Jenkins' assumptions are intermediate to ours. Although the authors agreed that selection of the progression parameters (which determine the duration of stages) was not unique, they did not vary the progression rate of HPV infection and therefore did not describe the complete range of the possible (cost-)effectiveness of HPV screening.

To explore the impact of longitudinal data, we simulated an observational cohort study with the two model versions A and B. In the simulation, women who entered the study with negative cytology have a Pap smear 5 years later. Predicted CIN detection rates in women who at entry were HPV negative and those who were HPV positive were discerned (Figure 2). As the description of cervical neoplasia (CIN and invasive cervical cancer) of the model was the same in both model versions, the detection rate for CIN at Pap smear screening 5 years after negative cytology was the same. In version A, however, almost 70% of the women with histologically confirmed CIN (low and high grade) came from previously HPV-positive women, whereas in model version B this was only 20%. This reflects a higher predictive value for future CIN of a positive HPV test in version A. The fact that longitudinal outcomes clearly differ in both models means that different longitudinal outcomes can be consistent with present cross-sectional data and that, once such longitudinal data are available, at least one (and probably both) of models A and B can be rejected. The range of combinations of parameter values on duration of HPV infection and sensitivity of the HPV test that are compatible with observed data will strongly decrease, and better predictions can be made of results expected from HPV screening.

Although the cross-sectional data show a strong association between HPV and cervical neoplasia, the results are insufficient to arrive at recommendations on screening. The discussion, therefore, on the representativeness of the test-positive rates that we aimed at in our simulation (4% in cytologically negative women, 67% in women with CIN and from 83% to 95% in women with invasive cervical cancers) is premature. Nonetheless, it is interesting to assess the influence of lower or higher observed HPV test-positive rates. In women with invasive cancer, the higher the HPV positiveness, the better this will be for the effectiveness of HPV screening. Higher test-positive rates in women with normal cytology and in women with CIN, however, can only mean that more women who do not develop cervical cancer will be HPV positive (all women that will develop HPV-positive cervical cancer are already assumed to be HPV positive before the development of the cancer). These women will unnecessarily be detected and followed up, and the negative side-effects and cost of follow-up will increase. In other words, given that HPV infection precedes, for example, 95% of the progressive neoplasias, lower HPV prevalence in the cytologically negative women and in women with CIN implies less harmless and less costly HPV screening.

A modelling approach, as presented in this paper, is useful for a joint analysis of cross-sectional, longitudinal and other relevant epidemiological data. We will adjust our model as soon as new evidence becomes available.

Data from large PCR-based cohort studies will accumulate in the forthcoming years. The fact that many of them are solely focused on young women should be of major concern. The Copenhagen study (Kjear et al, 1996) is restricted to women under 30 years of age, and the median age of the women in the Portland study is 34 years (Bauer et al, 1993). Screening for HPV in very young women would cause many women to be followed-up (because of the high prevalence in this age group of HPV infections that will clear) and is therefore not advisable. Moreover, the

© Cancer Research Campaign 1997

fact that prevalence is so much higher in younger age groups is also an expression of a different natural history of the HPV infection (at least a higher clearance rate) in this age group. Follow-up results from these women are obviously not transferable to the older age groups. Hence, further cohort studies should aim at women aged 30–60 years.

### ACKNOWLEDGEMENT

This study was financed by the Dutch Health Insurance Council.

### REFERENCES

Ballegooijen M Van, Habbema JDF, Oortmarssen GJ Van, Koopmanschap MA, Lubbe JTHN and Agt HMA Van (1992*a*) Preventive pap-smears: balancing costs, risks and benefits. *Br J Cancer* **65**: 930–933

Ballegooijen M Van, Koopmanschap MA, Subandono Tjokrowrdojo AJ and Oortmarssen GJ Van (1992*b*) Care and costs for advanced cervical cancer. *Eur J Cancer* **28A**: 1703–1708

Ballegooijen M Van, Koopmanschap MA and Habbema JDF (1995) The management of cervical intraepithelial neoplasia: extensiveness and costs. *Eur J Cancer* **13A**: 1672–1676

Bauer HM, Hildesheim A, Schiffman MH, Glass AG, Rush BB, Scott DR, Cadell DM, Kurman RJ and Manos MM (1993) Determinants of genital Human Papillomavirus infection in low-risk women in Portland, Oregon. *Sexually Transmitted Diseases* **20**: 274–278

Bergeron C, Barrasso R, Beaudenon S, Flamant P, Croissant O and Orth G (1992) Human papillomaviruses associated with cervical intra-epithelial neoplasia. *Am J Surg Pathol* **16**: 641–649

Bosch FX, Manos MM, Munoz N, Sherman M, Jansen AM, Peto J, Schiffman MH, Moreno V, Kurman R and Shah KV (1995) Prevalence of human papillomavirus in cervical cancer: a worldwide perspective. *J Natl Cancer Inst* **87**: 796–802

Brule AJC van den, Walboomers JMM, Maine M du, Kenemans P and Meijer CJLM (1991) Difference in prevalence of human papillomavirus genotypes in cytomorphological normal cervical smears is associated with a history of cervical intraepithelial neoplasia. *Int J Cancer* **48**: 404–408

Cuzick J, Terry G, Ho L, Hollingworth T and Anderson M (1994) Type-specific human papillomavirus DNA in abnormal smears as a predictor of high-grade cervical intraepithelial neoplasia. *Br J Cancer* **69**: 167–171

Cuzick J, Szarewski A, Terry G, Ho L, Hanby A, Maddox P, Anderson M, Kocjan G, Steele ST and Guilleband J (1995) Human papillomavirus testing in primary cervical cancer screening. *Lancet* **345**: 1533–1536

Dutch Network and National Database for Pathology (PALGA) (1992) *Results of Retrieval Action on Cervical Cytology from 1987–1990*. SIG: Utrecht

Dutch Network and National Database for Pathology (PALGA) (1995) *Results of Retrieval Action on Cervical Cytology from 1987–1990*. SIG: Utrecht

Eluf-Neto J, Booth M, Muñoz N, Bosch FX, Meijer CJLM and Walboomers JMM (1994) Human papillomavirus and invasive cervical cancer in Brazil. *Br J Cancer* **69**: 114–119

Gaarenstroom KN, Melkert P, Walboomers MM, Brule AJC van den, Bommel PFJ van, Meyer CJLM, Voorhorst FJ, Kenemans P and Helmerhorst THJM (1994) Human papillomavirus DNA and genotypes: prognostic factors for progression of cervical intraepithelial neoplasia. *Int J Gynaecol Cancer* **4**: 73–78

Ho GYF, Burk RD, Klein S, Kadish AS, Chang CJ, Palan P, Basu J, Tachezy R, Lewis R and Romney S (1995) Persistent genital human papillomavirus infection as a risk factor for persistent cervical dysplasia. *J Natl Cancer Inst* **87**: 1365–1371

IARC Working Group on Evaluation of Cervical Cancer Screening Programmes (1986) Screening for squamous cervical cancer: duration of low risk after negative results of cervical cytology and its implication for screening policies. *Br Med J* **293**: 659–664

IARC Working Group (1995) *IARC Monographs on the Evaluation of Carcinogenic Risks to Humans. Human Papilloma Viruses*. IARC Scientific Publication no. 64. IARC: Lyon

Jensen OM, Esteve J, Moller H and Renard H (1990) Cancer in the European community and its member states. *Eur J Cancer* **26**: 1167–1256

Jenkins D, Sherlaw-Johnson C and Gallivan S (1996) Can papilloma virus testing be used to improve cervical cancer screening? *Int J Cancer* **65**: 768–773

Kjaer SK, Brule AJC van den, Bock JE, Poll PA, Engholm G, Sherman ME, Walboomers JMM and Meijer CJLM (1996) Human papillomavirus – the most significant risk determinant of cervical intraepithelial neoplasia. *Int J Cancer* **65**: 601–606

Koopmanschap M, Lubbe JTHN, Oortmarssen GJ Van, Agt HMA van, Ballegooijen M Van and Habbema JDF (1990*a*) Economic aspects of cervical cancer screening. *Soc Sci Med* **30**: 1081–1087

Koopmanschap M, Oortmarssen GJ van, Agt HMA van, Ballegooijen M van, Habbema JDF and Lubbe JTHN (1990*b*) Cervical-cancer screening: attendance and cost-effectiveness. *Int J Cancer* **45**: 410–415

Lorincz AT, Reid R, Jenson AB, Greenberg MD, Lancaster WD and Kurman RJ. (1992) Human papillomavirus infection of the cervix: relative risk associations of fifteen common anogenital types. *Obstet Gynecol* **79**: 328–337

Melkert PWJ, Hopman E, Brule AJC van den, Risse EKJ, Diest PJ van, Bleker OP, Helmerhorst T, Schipper MEI, Meijer CJLM and Walboomers JMM (1993) Prevalence of HPV in cytomorphological normal cervical scrapes, as determined by the polymerase chain reaction, is age-dependent. *Int J Cancer* **53**: 919–923

Morrison EAB, Ho GYF, Vermund SH, Goldberg GL, Kadish AS, Kelley KF and Burk RD (1991) Human papillomavirus infection and other risk factors for cervical neoplasia: a case–control study. *Int J Cancer* **49**: 6–13

Muñoz N, Bosch FX, Sanjosé S de, Tafur L, Izarzugaza I, Gili M, Viladiu P, Navarro C, Martos C, Ascunce N, Gonzalez LC, Kaldor JM, Guerrero E, Lörincz A, Santamaria M, Alfonso de Ruiz P, Aristizabal N and Shah K (1992) The causal link between human papillomavirus and invasive cervical cancer: a population-based case–control study in Colombia and Spain. *Int J Cancer* **52**: 743–749

Oortmarssen GJ van and Habbema JDF (1991) Epidemiologic evidence for age-dependent regression of pre-invasive cervical cancer. *Br J Cancer* **64**: 559–565

Oortmarssen GJ Van and Habbema JDF (1995) The duration of pre-clinical cancer and the reduction in incidence of invasive cancer following negative Pap smears. *Int J Epidemiol* **24**: 300–307

Parkin DM, Pisani P and Ferlay J (1993) Estimates of the worldwide incidence of eighteen major cancers in 1985. *Int J Cancer* **54**: 954–606

Remmink AJ, Walboomers JMM, Helmerhorst TJM, Voorhorst FJ, Rozendaal L, Risse EKJ, Meijer CJLM and Kenemans P (1995) The presence of persistent high risk HPV genotypes in dysplastic cervical lesions is associated with progressive disease: natural history up to 36 months. *Int J Cancer* **61**: 306–311

Rozendaal L, Walboomers JMM, Linden JC van der, Voorhorst FJ, Kenemans P, Helmerhorst THJM, Ballegooijen M van and Meijer CJLM (1996) The PCR-based high risk HPV test in cervical cancer screening gives an objective risk assessment of women with cytomorphological normal cervical smears. *Int J Cancer* **68**: 766–769

Sanjosé S de, Muñoz N, Bosch FX, Reimann K, Pedersen NS, Orfila J, Ascune N, Gonzalez LC, Tafur L, Gili M, Lette I, Viladiu P, Tormo MJ, Moreo P, Shah K and Wahren B (1994) Sexually transmitted agents and cervical neoplasia in Colombia and Spain. *Int J Cancer* **56**: 358–363

Walboomers JMM, Roda Husman AM de, Snijders PJF, Stel HV, Risse EKJ, Helmerhorst TJM, Voorhorst FJ and Meijer CJLM (1995) Human papillomavirus in false negative archival cervical smears: implications for screening for cervical cancer. *J Clin Pathol* **48**: 728–732

Zur Hausen H (1994) Molecular pathogenesis of cancer of the cervix and its causation by specific human papillomavirus types. In *Human Pathogenic Papillomaviruses*, Zur Hausen H. (ed.), pp. 9–13. Springer: Berlin

© Cancer Research Campaign 1997